# Lysophosphatidylcholine and lysophosphatidic acid as key messengers in atherosclerosis: from a lipid-inflammation vicious cycle to therapeutic translation

**DOI:** 10.3389/fcvm.2026.1875638

**Published:** 2026-07-21

**Authors:** Xue Guan, Zhongyan Li, Huan Cheng, Jingru Li, Najie Wen, Luqiao Wang

**Affiliations:** Department of Cardiology, The First Affiliated Hospital of Kunming Medical University, Kunming, Yunnan, China

**Keywords:** atherosclerosis, lipid inflammation vicious cycle, lysophospholipids, macrophage pyroptosis, spatial metabolomics

## Abstract

The residual risk of atherosclerosis (AS) extends beyond low-density lipoprotein cholesterol (LDL-C) and involves a complex interplay between lipid metabolism and inflammation. Among lysophospholipids (LPLs), lysophosphatidylcholine (LPC) and lysophosphatidic acid (LPA), the two most abundant and bioactive LPL species in AS plaques, serve as central hubs in this interaction. Hyperlipidemia provides the substrate for LDL oxidation; the resulting oxidized LDL is hydrolyzed by lipoprotein-associated phospholipase A_2_ (Lp-PLA_2_) to generate LPC, which is further converted by autotaxin to the more potent LPA. These LPLs activate specific G protein-coupled receptors (GPCRs) on endothelial cells, macrophages, and vascular smooth muscle cells, thereby triggering inflammatory signaling, impairing reverse cholesterol transport, and promoting macrophage pyroptosis. Spatial metabolomics has identified LPC (18:0) and LPA (18:1) as subspecies enriched in macrophage-rich regions of vulnerable plaques, providing direct *in-situ* evidence for their pathogenic roles. This review systematically examines the biosynthesis, receptor-mediated signaling, and pathological effects of LPC and LPA in AS, with an emphasis on the LPC-LPA axis as a self-reinforcing driver of plaque progression and destabilization. We further critically evaluate translational advances, including failed clinical trials of Lp-PLA_2_ and autotaxin inhibitors, and discuss emerging strategies such as multi-node combination therapy and subtype-specific precision targeting of LPLs. By integrating mechanistic insights with spatial metabolomics and therapeutic perspectives, this review aims to inform future strategies to overcome the residual risk of AS.

## 1 Introduction

Atherosclerosis (AS) is a chronic disease of large- and medium-sized arteries. Its pathological foundation consists of abnormal lipid accumulation in the intima, chronic inflammation, extracellular matrix remodeling, and plaque formation. Plaque growth can narrow the vessel lumen; plaque rupture leads to acute thrombotic events such as myocardial infarction and ischemic stroke. These features make AS the leading cause of cardiovascular death and disability worldwide (1, 2). According to the latest Global Burden of Disease projections, between 2025 and 2050, the global prevalence of cardiovascular disease will rise by 90.0%, and the crude death rate by 73.4%. By 2050, annual CVD deaths will rise from 20.5 million in 2025 to 35.6 million (3). This growing disease burden is driven primarily by atherosclerotic disease (3).

Even though statins and other lipid-lowering therapies have substantially reduced cardiovascular risk, many patients still carry a considerable “residual risk”. For example, approximately one in five survivors of acute coronary syndrome experiences another cardiovascular event within five years despite optimized medical therapy (4). PCSK9 inhibitors produce additional reductions in LDL cholesterol (LDL-C), but their clinical benefit varies markedly across individuals. The median estimated lifetime treatment gain is just five months, and 61% of patients derive less than six months of clinical benefit (5). This finding underscores that mechanisms beyond LDL-C play a critical role in AS progression.

Over the past decades, the view of AS has shifted from a “lipid storage disease” to a “chronic inflammatory disease”. It is now widely accepted that lipid metabolism disorders and immune-inflammatory responses are closely linked (6, 7). Nevertheless, a key question remains unresolved: how is abnormal lipid metabolism “translated” into the inflammatory language of the vessel wall? Within this complex signaling network, lysophospholipids (LPLs) have garnered growing research attention. This class of lipid signaling molecules is generated from membrane phospholipids via the action of phospholipases.

Among the various LPLs, lysophosphatidylcholine (LPC) and lysophosphatidic acid (LPA) stand out as the most studied ones—they are abundant in AS plaques, highly bioactive, and deeply intertwined with both lipid metabolism and inflammatory signaling (8–11). LPC is the most abundant LPL in plasma (>100 μmol/L). It is generated mainly by lipoprotein-associated phospholipase A_2_ (Lp-PLA_2_) hydrolyzing phosphatidylcholine on oxidized LDL (ox-LDL), and acts as an “initiating messenger” that turns on endothelial inflammation (10, 12–14). LPA, in turn, is converted from LPC by autotaxin (ATX) via its lysophospholipase D activity; it is more potent and serves as an “amplifying messenger” that regulates multiple vascular wall cell types through G protein-coupled receptor (GPCR) networks (11, 15, 16).

Accumulating evidence indicates that LPLs are not passive byproducts of atherosclerotic disease. Instead, they actively transduce upstream lipid disturbances and drive downstream inflammatory cascades, giving rise to a self-reinforcing pathogenic cycle. Hyperlipidemia supplies the LDL substrate; oxidative stress acts as the switch that converts LDL into ox-LDL; Lp-PLA₂ and ATX then sequentially generate LPC and LPA. These lipid messengers drive inflammatory signaling via their receptor networks and induce macrophage pyroptosis. In turn, they impair reverse cholesterol transport and exacerbate local lipid accumulation, creating a positive feedback loop that accelerates lesion progression (17, 18). Spatial metabolomics, using matrix-assisted laser desorption/ionization mass spectrometry imaging (MALDI-MSI), has mapped lipid molecules in aortic root plaques of ApoE⁻/⁻ mice. It identified for the first time a marked enrichment of LPC (18:0) and LPA (18:1) in macrophage-rich areas of advanced plaques and confirmed these findings in human carotid atherosclerotic plaques, where the same LPL subspecies were found to be concentrated in macrophage-rich shoulder regions and necrotic cores (18). Moreover, plasma levels of these two lipids are significantly associated with future cardiovascular risk. This observation provides direct *in situ* molecular evidence for the pathogenic cycle outlined above (18).

**Figure 1:**
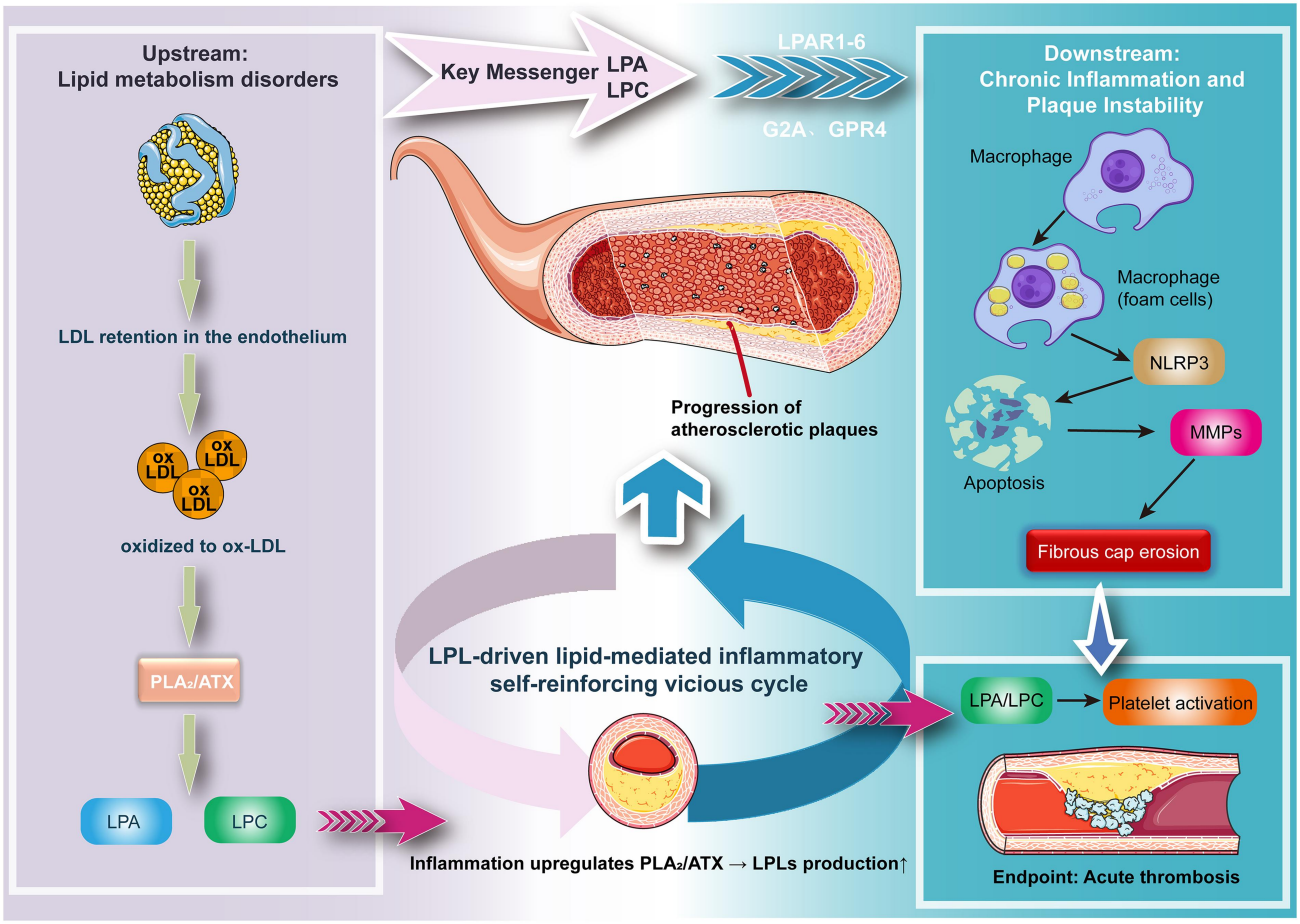
The lipid-inflammation pathogenic cycle by which LPLs act as key messengers driving atherosclerosis progression. LPLs (primarily LPA and LPC) serve as key messengers bridging upstream lipid metabolism disorders and downstream chronic inflammation in AS, forming a self-reinforcing pathogenic cycle that promotes plaque progression, destabilization, and ultimately triggers acute thrombotic events. As shown on the left, LDL is retained in the vascular endothelium and oxidized to ox-LDL, which is then catalyzed by PLA₂/ATX to generate LPA and LPC—completing “messenger production”. These LPLs transmit signals through specific receptors (LPAR_1−6_, G2A/GPR4) on endothelial cells, macrophages, and smooth muscle cells, driving macrophage-to-foam-cell transformation, NLRP3 inflammasome activation and pyroptosis, MMP release, and fibrous cap erosion, leading to plaque instability (right-hand pathways). The local inflammatory microenvironment in the plaque can further upregulate PLA_2_/ATX expression, promoting even more LPL production and creating a positive feedback loop of “lipid disturbance → LPL generation → inflammation activation → more LPL generation” (circular arrows at the bottom). Finally, after vulnerable plaque rupture, the exposed LPA/LPC directly activate platelets, triggering acute thrombosis and clinical adverse events.

Against this background, this review uses the “lipid-inflammation pathogenic cycle” as its central organizing framework. We systematically examine the biosynthesis of LPC and LPA, their receptor-mediated signaling, and the pathological effects they exert across the course of AS, with particular emphasis on LPL-driven macrophage pyroptosis in plaque destabilization. We also integrate spatial metabolomics data with ongoing therapeutic translation efforts and propose multi-node combination strategies. These are coordinated interventions that concurrently target three key levels: reducing upstream lipid substrates, modulating midstream LPL signaling, and blocking downstream inflammatory effector pathways, with the goal of achieving more effective disruption of the LPL network. The core pathways of this cycle are illustrated in Figure 1.

## 2 Initiation of the vicious cycle: production and signal decoding of LPLs

LPLs act as messengers between lipid metabolism and inflammation. Their biological effects depend on a complete “production-relay-decoding” system. Understanding this system's molecular architecture is essential for uncovering how they drive AS pathology. This chapter focuses on the specific pathways that generate LPC and LPA in the AS plaque microenvironment, and on how these lipids use key receptors to turn extracellular lipid signals into intracellular pathological responses.

### 2.1 Biosynthesis of LPC and LPA: from lipid substrates to active messengers

In the context of AS, the production of LPC and LPA is not random. It is a cascade driven by both hyperlipidemia and oxidative stress. Under hyperlipidemic conditions, large numbers of LDL particles cross the damaged endothelium and become trapped in the intima (19–21). AS begins with the subendothelial retention of apolipoprotein B (apoB)-containing lipoproteins in the extracellular matrix and proteoglycans of the arterial wall; trapped LDL then undergoes a series of pro-inflammatory modifications within the intimal microenvironment (22–24). This relatively closed microenvironment is rich in reactive oxygen species (ROS), which oxidize LDL to ox-LDL (25–27). Ox-LDL generated under oxidative stress drives atherosclerosis by impairing endothelial function, promoting foam cell formation, and triggering vascular inflammation (28, 29). The phosphatidylcholine (PC) on the surface of oxidized low-density lipoprotein (ox-LDL) is readily susceptible to hydrolysis, and lipoprotein-associated phospholipase A_2_ (Lp-PLA_2_) plays a central role in this reaction. By hydrolyzing oxidized phosphatidylcholine (oxPC) to produce lysophosphatidylcholine (lysoPC), this enzyme constitutes the primary source of LPC within atherosclerotic plaques (30).

LPC is not the end of this cascade. Locally in the vessel wall, LPC is further metabolized by the secreted enzyme ATX into the more potent LPA (11, 31, 32). ATX is a secreted glycoprotein whose lysophospholipase D (lysoPLD) activity is the major rate-limiting step for extracellular LPA production, and its expression is markedly upregulated at sites of inflammation and tissue injury (10, 11, 33). The ATX-LPA axis acts as a “signal amplifier” for the conversion of LPC to LPA: LPC, the initiating signal, is catalyzed by ATX into the more active LPA, which then amplifies the local lipid disturbance signal and transmits it to multiple effector cell types (34, 35). Because this conversion chain is intact, targeting ATX may not only block LPA generation but also reduce the feedback consumption of LPC, thereby downregulating LPL signaling from both directions (36, 37). Beyond LDL, lipoprotein(a) [Lp(a)] serves as an additional and functionally important carrier of LPLs in the circulation. Lp(a) not only transports oxidized phospholipids with a high content of LPC but also preferentially associates with ATX, the enzyme responsible for converting LPC to LPA. Studies have demonstrated that ATX activity is significantly higher in Lp(a) fractions than in LDL fractions, and that ATX-Lp(a) complexes can be detected in human plasma (38). This ATX-Lp(a) complex is functionally active, allowing targeted delivery of LPA to specific vascular sites.

The pathophysiological relevance of this Lp(a)-ATX-LPA axis is particularly evident in calcific aortic valve stenosis (CAVS). Lp(a)-derived ATX, together with ATX produced locally by valve interstitial cells, promotes inflammation and mineralization of the aortic valve through LPA-mediated signaling via the NF-*κ*B/IL-6/bone morphogenetic protein pathway (39). In patients with CAVS, ATX-Lp(a) levels are significantly associated with disease presence independent of conventional risk factors (38). Furthermore, recent lipidomic studies have revealed that Lp(a) particles are enriched not only in LPC but also in LPA itself and other pro-inflammatory lipids such as diacylglycerols (DGs) (40). Lp(a)-associated DGs activate monocytes via the NLRP3 inflammasome, leading to secretion of IL-6, IL-8, and IL-1β, while LPA promotes monocyte transendothelial migration (40). In human atherosclerotic plaques, high Lp(a) levels correlate with the presence of pro-inflammatory macrophage subsets and Lp(a) colocalization with M1 macrophages (40). Collectively, these findings establish Lp(a) as a critical upstream carrier that delivers LPC, ATX, and LPA directly to the vessel wall, thereby amplifying the LPL-driven inflammatory cascade beyond what LDL alone can provide.

### 2.2 Receptor decoding: from extracellular signal to intracellular pathology

The biological effects of LPC and LPA depend on signal transduction through seven-transmembrane G protein-coupled receptors (GPCRs) on the cell surface and through nuclear receptors (41, 42). Rather than giving a textbook-style list of pathways, this section only outlines the core receptor framework that supports the “lipid-inflammation cycle”. The detailed pathway functions will be integrated with pathology in the next chapter.

The main receptors for LPC include G2A (GPR132) and GPR4 (43, 44). Li et al. (41) systematically reviewed the roles of lysophospholipids and their GPCRs in AS, and noted that G2A mainly couples to G*α*i/o and G*α*_12/13_, activates MAPK (ERK, p38) and RhoA/ROCK pathways, induces endothelial cell apoptosis, and promotes VSMC migration. GPR4 engages similar pro-inflammatory signaling cascades. In addition, Liu et al. (45) showed that LPC activates the NLRP3 inflammasome in brain microvascular endothelial cells through a GPR4-mediated pathway, and that GPR4 silencing markedly weakens the pro-inflammatory effect of LPC. Beyond GPCRs, LPC also activates the nuclear receptor PPAR*γ*—Wang et al. (46) demonstrated that LPC has PPAR*γ* agonist activity, and the classic study by Tontonoz et al. (47) showed that PPAR*γ* promotes monocyte/macrophage differentiation and ox-LDL uptake. Rahaman et al. (48) further established that a CD36-dependent signaling cascade is required for foam cell formation. LPC exerts its effects through a dual decoding system. G2A/GPR4 mediates the transduction of inflammatory signals, whereas PPAR*γ* regulates lipid uptake. This two-tiered regulatory mechanism establishes LPC as an initiating messenger that triggers the inflammatory program within the vessel wall.

LPA acts through six high-affinity receptors (LPAR_1–6_) (11, 15). Li et al. (41) point out that these receptors couple to different G proteins and activate multiple downstream pathways such as PI3 K/Akt, Ras/MAPK, and RhoA/ROCK. In AS plaques, LPAR_1_ largely co-localizes with smooth muscle cell markers and drives VSMC proliferation and migration; LPAR₂ and LPAR₆ are more associated with macrophages and endothelial cells, and participate in building a pro-inflammatory microenvironment (49). Pei et al. (50) clearly showed that LPA_2_ helps maintain vascular endothelial homeostasis after myocardial infarction, supporting its role in endothelial dysfunction. The diverse repertoire of LPA receptors underpins its core role as an amplifying messenger. It converts the local signal triggered by LPC into a systemic pathological cascade that engages multiple cell types (16). Table 1 summarizes the core receptors, downstream pathways, and pathological effects of LPC and LPA.

**Table 1:** Key receptors, downstream signaling pathways, and pathological effects of LPC and LPA in atherosclerosis.

Lipid	Receptor	Primary cell type(s) in AS	Downstream pathways	Pathological effects
LPC	G2A (GPR132)	Endothelial cells, macrophages (inferred from non-AS models)	MAPK (ERK, p38), RhoA/ROCK	Endothelial apoptosis, upregulation of VCAM-1/ICAM-1, monocyte adhesion
	GPR4	Endothelial cells (demonstrated in non-AS models	NLRP3 inflammasome activation (demonstrated in endothelial cells)	IL-1*β* release, pyroptosis
	PPAR*γ*	Macrophages	Nuclear receptor activation, lipid uptake gene transcription	Foam cell formation, monocyte/macrophage differentiation
LPA	LPAR1	VSMCs	RhoA/ROCK	VSMC proliferation, migration, contractile-to-synthetic phenotype switching
	LPAR2	Endothelial cells, macrophages	PI3 K/Akt, PLC-Raf1-Erk	Angiogenesis, endothelial dysfunction, pro-inflammatory cytokine secretion
	LPAR6	VSMCs (presumed)	Not fully characterized in AS	Potentially pro-inflammatory; may modulate foam cell formation
	LPAR3	Macrophages, endothelial cells	RhoA/ROCK (presumed)	Likely similar to LPAR1

The cell-type-specific distribution of LPC and LPA receptors is central to their pathological functions in AS. As summarized in Table 1, LPC primarily engages G2A on endothelial cells to promote adhesion molecule expression and monocyte recruitment, while GPR4-mediated NLRP3 activation has been demonstrated in endothelial cells. PPARγ, activated by LPC in macrophages, drives foam cell formation and monocyte differentiation. For LPA, LPAR1 is predominantly expressed on VSMCs, where it induces proliferation and migration; LPAR2 is enriched on endothelial cells and macrophages, promoting angiogenesis and inflammatory cytokine secretion; LPAR6 is also associated with macrophages and endothelial cells, though its role in AS remains incompletely characterized. This receptor–cell type mapping highlights the multi-cellular nature of LPL-driven pathology and underscores the importance of cell-type-specific targeting strategies.

G2A/GPR132, G protein-coupled receptor 132; GPR4, G protein-coupled receptor 4; MAPK, mitogen-activated protein kinase; RhoA, Ras homolog family member A; ROCK, rho-associated coiled-coil containing protein kinase; VCAM-1, vascular cell adhesion molecule-1; ICAM-1, intercellular adhesion molecule-1; NLRP3, NLR family pyrin domain containing 3; IL-1β, interleukin-1β; PPARγ, peroxisome proliferator-activated receptor γ; LPAR, lysophosphatidic acid receptor; VSMC, vascular smooth muscle cell; PI3 K, phosphoinositide 3-kinase; PLC, phospholipase C; Raf1, rapidly accelerated fibrosarcoma 1 kinase; ERK, extracellular signal-regulated kinase.

## 3 Vicious cycle in action: pathological effects of LPLs

The core value of LPLs as “messengers” is that they translate upstream lipid disturbance signals into specific pathological events in the vessel wall, step by step. This chapter is organized along the temporal logic of “initiation → amplification → terminal progression”, and examines how LPC and LPA act in concert to drive the full pathological sequence of AS. This cascade spans from early endothelial inflammation through to acute thrombosis.

### 3.1 Initiation: LPC triggers endothelial inflammation and monocyte recruitment

In early AS, LPC generated by Lp-PLA_2_ hydrolysis of ox-LDL is a key molecular event that initiates endothelial dysfunction (51). Although ox-LDL can directly activate endothelial cells via multiple receptor pathways, including LOX-1, TLR4, and CD36, LPC remains a critical downstream mediator. It accounts for a substantial proportion of the pro-inflammatory effects exerted by ox-LDL (52, 53). Kougias et al. (51) demonstrated that LPC and sPLA₂ are key mediators of endothelial dysfunction and atherosclerosis. More recent work has confirmed that LPC activates NADPH oxidase 5 (NOX5) in endothelial cells, boosting ROS production and triggering oxidative stress. This oxidative stress inflicts direct damage on endothelial cells and suppresses the activity of endothelial nitric oxide synthase (eNOS), which in turn impairs vasodilatory function. This functional impairment represents one of the earliest hallmarks of atherosclerotic disease (54, 55).

More importantly, LPC acts through the G2A receptor to activate p38-MAPK and other signaling pathways, inducing endothelial cell apoptosis and upregulating vascular cell adhesion molecule-1 (VCAM-1) and intercellular adhesion molecule-1 (ICAM-1) (56). Higher levels of these adhesion molecules strongly promote the adhesion of circulating monocytes and other immune cells to the endothelium and their subsequent transendothelial migration, setting the stage for inflammation and lipid plaque formation (57–59). In this process, LPC acts as a critical messenger for ox-LDL. It translates the upstream signal of lipid dysregulation into downstream cellular responses that initiate the inflammatory recruitment program.

### 3.2 Amplification: LPA extends signaling to multicellular systemic pathological responses

Locally in the vessel wall, LPC is further metabolized by efficiently secreted ATX into the more potent LPA (60, 61). As an “amplifying messenger”, LPA binds to six different GPCRs (LPAR_1–6_) on the cell surface, greatly amplifying the pathogenic effects of LPLs (62, 63). Expression profiling of human AS plaques has revealed that LPARs are widely expressed across multiple cell types within the plaque: LPAR_1_ co-localizes with smooth muscle cell markers, whereas LPAR₂ and LPAR_6_ are more associated with macrophages and endothelial cells, suggesting differential functions of receptor subtypes in AS (49).

Specifically, LPA strongly induces VSMC proliferation and migration through the LPAR_1/3_-G*α*_12/13_-RhoA/ROCK pathway, driving their switch from a contractile to a synthetic phenotype (61). In vascular endothelial cells, the LPA-LPAR_2_ pathway promotes proliferation and tube formation via PI3K-Akt and PLC-Raf1-Erk, thereby enhancing angiogenesis (50). At the same time, through LPAR-mediated signals, LPA acts directly on macrophages to inhibit cholesterol efflux, thus promoting foam cell formation (64). While LPC drives the initiation of this pathogenic cascade, LPA acts as the core amplifying mediator, scaling local LPC-initiated signals into a systemic pathological response across multiple cell types.

Together, LPA and LPC create a strong local pro-inflammatory microenvironment (10, 65, 66). LPA activates p38 MAPK and PKC signaling through the LPA1 receptor, inducing vascular smooth muscle cells (VSMCs) to secrete interleukin-6 (IL-6) and monocyte chemoattractant protein-1 (MCP-1) (67). LPC, via its receptors, activates MAPK and other pathways, inducing various inflammatory cytokines and chemokines in endothelial cells and macrophages (10, 66). In addition, LPC directly activates the NLRP3 inflammasome in macrophages, triggering IL-1*β* secretion and pyroptosis (10, 65), thereby driving plaque destabilization (68).

### 3.3 Terminal phase: LPLs activate platelets and promote acute thrombosis

During the long course of AS, LPLs not only contribute to chronic plaque progression but also play a key role in acute thrombosis after plaque erosion or rupture (61). LPC is a core component of platelet microvesicles derived from unstable plaques and can directly activate platelets and promote their aggregation (69). In addition, LPA itself is a potent platelet activator. Through LPAR family receptors (especially LPAR_1_), it activates PI3 K/Akt and p38 MAPK pathways inside platelets, leading to shape change, granule release, and fibrinogen receptor activation (70, 71). At the moment of plaque rupture, the exposed lipid core, rich in LPA and LPC, rapidly activates platelets and the coagulation system, triggering acute thrombosis (61). In the terminal phase of this pathogenic cascade, LPLs once again act as signaling messengers. They transduce structural damage signals from ruptured plaques into acute vascular occlusion via rapid thrombus formation, a process that directly determines patients' clinical outcomes.

## 4 Terminal of the vicious cycle: macrophage pyroptosis and plaque destabilization

Far from only inducing functional disturbances, LPLs directly mediate structural plaque damage through triggering macrophage pyroptosis. This is a major breakthrough in recent AS mechanism research and the most representative example of how LPLs, as key messengers, convert lipid signals into tissue injury.

### 4.1 Molecular pathway of LPC-induced NLRP3 inflammasome activation

LPC, as the main active component of ox-LDL, is a potent endogenous stimulator that activates the NLRP3 inflammasome in macrophages. Corrêa et al. (65) showed that LPC induces NLRP3 inflammasome-dependent pyroptosis in human monocytes and endothelial cells, and promotes foam cell formation. Pharmacological inhibition assays show that LPC mediates IL-1*β* release and inflammasome activation through potassium efflux and lysosomal damage. Both glyburide (a potassium efflux inhibitor) and CA-074 (cathepsin B inhibitor targeting lysosomal damage) significantly reduce LPC-induced IL-1*β* secretion (65). However, that study did not identify the upstream receptor(s) by which LPC activates NLRP3; it only suggested that lysosomal damage and potassium efflux serve as key second signals.

At the receptor level, the involvement of LPC's main receptors, G2A (GPR132) and GPR4, in NLRP3 activation has been supported by other studies, although most of the evidence comes from non-AS models or non-macrophage cell types. Liu et al. (45) showed that LPC activates the NLRP3 inflammasome in brain microvascular endothelial cells through a GPR4-mediated pathway, and that GPR4 silencing markedly reduces the pro-inflammatory effect of LPC. Freeman et al. (72) demonstrated in a demyelination model that LPC, acting as a damage-associated molecular pattern, activates NLRP3 and NLRC4 inflammasomes in microglia and astrocytes via the G2A receptor. This process requires ASC, caspase-1, cathepsin-mediated degradation, calcium mobilization, and potassium efflux; Nlrp3⁻/⁻Nlrc4⁻/⁻ double-knockout mice showed the most pronounced reduction in astrocyte proliferation and microglial accumulation, accompanied by decreased G2A expression (72).

It should be noted that the proposed G2A/GPR4 → NLRP3 axis in AS plaque macrophages remains inferential and lacks direct validation. Current evidence for this pathway comes primarily from brain microvascular endothelial cells (for GPR4) and a demyelination model (for G2A), neither of which fully recapitulates the atherosclerotic plaque microenvironment. Whether LPC activates the NLRP3 inflammasome in AS plaque macrophages via G2A or GPR4, and whether these receptors contribute to pyroptosis and plaque destabilization *in vivo*, have not been directly demonstrated. Future studies using macrophage-specific knockout mice and AS-relevant stimuli are needed to establish causality and determine the relative contribution of each receptor.

Taken together, the most likely pathway for LPC to activate the NLRP3 inflammasome is as follows: LPC triggers an intracellular signaling cascade through G2A/GPR4, leading to increased lysosomal membrane permeability and cathepsin B leakage, while also provoking potassium efflux. Together, these two events provide the second signal needed for NLRP3 inflammasome assembly and activation. That said, the exact receptor mechanism by which LPC activates NLRP3 in AS plaque macrophages is still unclear—current evidence comes mainly from brain microvascular endothelial cells (GPR4 studies) and a demyelination model (G2A studies). Future work needs to validate the direct G2A/GPR4→NLRP3 causality in primary macrophages and AS animal models.

### 4.2 Structural link between pyroptosis and plaque instability

Macrophage pyroptosis exerts multiple destructive effects on AS plaque instability. First, pyroptosis reduces the number of live macrophages within the plaque while releasing large amounts of inflammatory cytokines, creating a “cell death-inflammation amplification” pathogenic cycle (73). Second, macrophage pyroptosis constitutes a critical cellular basis for necrotic core expansion. Extensive pyroptotic death of foam cells directly drives the accumulation of lipid debris and elevates the inflammatory burden within the necrotic core (68, 74). Third, proteases (including matrix metalloproteinases, MMPs) and inflammatory mediators released by pyroptosis erode the collagen matrix of the fibrous cap, weakening its mechanical strength and thereby leading to plaque destabilization (75). Fourth, cytokines such as IL-1*β* further recruit circulating monocytes, sustaining and amplifying local inflammation (73).

In summary, LPLs translate upstream lipid metabolism disturbances directly into physical destruction of plaque structure by inducing macrophage pyroptosis. This is the core mechanism through which LPLs, as “messengers”, move from merely regulating function to actually executing tissue damage. LPC, the main active component of ox-LDL, triggers lysosomal damage and potassium efflux via G2A/GPR4, thereby activating the NLRP3 inflammasome-caspase-1-GSDMD axis, which drives macrophage pyroptosis and releases IL-1β and other inflammatory mediators. Thus, LPLs not only transmit lipid abnormality signals but also become direct executors of plaque structural breakdown. Targeting pyroptosis pathways (e.g., NLRP3 or IL-1β) holds promise for interrupting this terminal pathogenic cycle and offers a new strategy to stabilize vulnerable plaques.

## 5 Spatial metabolomics: direct tissue evidence of the lipid-inflammation cycle

The biochemical and cellular evidence discussed above has clarified the signaling mechanisms of LPLs in AS. However, a key question remains unanswered: conventional metabolomics based on tissue homogenates loses spatial information, so do these molecular events distribute in plaque tissue as expected? In recent years, spatial metabolomics techniques, exemplified by matrix-assisted laser desorption/ionization mass spectrometry imaging (MALDI-MSI), have enabled label-free, high-resolution visualization of specific lipid molecules directly in tissue sections, thereby providing direct anatomical evidence for the lipid-inflammation cycle (18, 76). MALDI-MSI achieves a favorable balance among sample preparation, chemical sensitivity, and spatial resolution, making it particularly suitable for analyzing the complex molecular heterogeneity of atherosclerotic lesions (77, 78). Currently, MALDI-MSI can reach a spatial resolution of 10 μm or even approach single-cell level, and can be coupled with high-resolution mass spectrometers for precise identification of lipid species (77, 78). These technological advances allow researchers to accurately map the molecular landscape of various LPL subspecies in aortic root plaques of classic AS models such as ApoE⁻/⁻ mice (79, 80).

### 5.1 Evidence from human carotid plaques: linking lipid distribution to plaque stability

Several studies have applied MALDI-MSI to human carotid plaques to investigate region-specific lipid alterations associated with clinical presentation. Greco et al. (81) compared symptomatic vs. asymptomatic plaques and found that macrophage-rich regions of symptomatic lesions exhibited significantly higher sphingomyelin, whereas intimal vascular smooth muscle cells showed increased cholesterol and cholesteryl esters. In asymptomatic plaques, smooth muscle cells in the fibrous cap were enriched in lysophosphatidylcholine and cholesteryl esters, both of which promote smooth muscle cell proliferation and migration, thereby favoring stable fibrous cap formation. Macrophage-rich regions of symptomatic lesions were enriched in phosphatidylcholine (synthesized to counteract excess free cholesterol), whereas the same regions in asymptomatic plaques were enriched in polyunsaturated cholesteryl esters and triglycerides, which are features of functional lipid droplets (81). The same team also established a multimodal MALDI-MSI workflow integrating histology and immunofluorescence, enabling precise co-localization of lipids with pathological structures (81).

Khamehgir-Silz et al. (76) compared the lipid profiles of ApoE⁻/⁻ mouse aortas with human atherosclerotic peripheral arteries. They identified 31 lipid species – including cholesteryl esters, lysophosphatidylcholines, lysophosphatidylethanolamines, and cholesterol derivatives – that were specifically enriched in aortic plaques of aged ApoE⁻/⁻ mice; 26 of these lipid markers were also detectable in human atherosclerotic peripheral arteries. Notably, glucosylated cholesterol species were identified as potential novel markers specific to human atherosclerotic vessels, whereas they were not detected in mouse tissues. This highlights species differences and calls for caution when translating findings from mouse models to clinical settings (76).

Beyond the above findings, recent spatial lipidomics studies using MALDI-MSI have further expanded the lipid landscape of atherosclerotic plaques. In a familial hypercholesterolemia swine model, Slijkhuis et al. (82) identified 473 lipid-related features and found that ether-linked phospholipids and LysoPC species colocalized with the necrotic core, while cholesteryl esters, ceramides, and phosphatidylinositol species colocalized with inflammatory cells (82). In a complementary human study, the same group applied DESI-MSI to human carotid plaques and visualized the spatial distribution of 611 lipid-related m/z features, of which 330 could be confidently assigned (83). Collectively, these studies reinforce the concept that LPLs and other lipid species are regionally concentrated in vulnerable plaque microenvironments, providing additional independent evidence for the lipid-inflammation cycle discussed in this review.

### 5.2 Landmark evidence: co-localization of LPC (18:0) and LPA (18:1) in vulnerable plaque regions

The strongest evidence linking LPLs to plaque instability comes from the landmark 2024 study by Cao et al. in *Arteriosclerosis, Thrombosis, and Vascular Biology* (18). Using MALDI-MSI on aortic root plaques from ApoE⁻/⁻ mice and validation in human carotid plaques, the study demonstrated that the signal intensities of LPC (18:0) and LPA (18:1) were highly co-localized in plaque regions, particularly enriched in macrophage-rich shoulder areas and the rupture-prone necrotic core. Quantitative analysis showed that both LPL candidate biomarkers were significantly elevated in advanced plaques compared with early plaques (both *P* < 0.05), and their plasma levels correlated significantly with future cardiovascular risk, providing direct *in-situ* molecular evidence for the pathogenic cycle. These specific LPL candidate biomarkers carrying saturated or monounsaturated fatty acyl chains likely play a predominant role in driving local inflammation, inducing cell death and necrosis, and promoting plaque destabilization.

Spatial metabolomics also helps identify therapeutic targets and predictive biomarkers. Cao et al. further demonstrated that plasma levels of LPC (18:0) and LPA (18:1) can identify patients at higher cardiovascular risk, suggesting their potential as circulating biomarkers for patient stratification in future clinical trials. Greco et al. (81) also identified lipid markers associated with human carotid plaque instability using mass spectrometry imaging. Collectively, these studies indicate that LPLs are not uniformly distributed across plaques but are concentrated in pathological micro-regions such as the shoulder and necrotic core. This aligns well with the lipid-inflammation cycle described earlier: LPL generation driven by hyperlipidemia does not spread indiscriminately but selectively enriches in the most vulnerable, highest-risk areas, forming self-reinforcing pathological centers.

### 5.3 Summary and outlook

In summary, spatial metabolomics has captured key molecular evidence of the lipid-inflammation cycle *in situ* at unprecedented resolution. Converging evidence from multiple independent studies establishes LPLs as core drivers of AS pathology. Key supporting findings include the enrichment of LPC (18:0) and LPA (18:1) in vulnerable plaques (18), region-specific lipid alterations associated with symptomatic disease (81), and the translational potential of these lipid signatures as plasma biomarkers. Taken together, these data provide a molecular foundation for precision medicine targeting LPL-mediated pathways. These advances offer clear anatomical coordinates for future precision therapies: interventions should avoid indiscriminate targeting of all LPL subspecies and instead focus on those specific subspecies that selectively accumulate in these pathological hotspots.

It should be noted that current spatial metabolomics data are mostly derived from cross-sectional studies or animal models, and there is still a lack of longitudinal follow-up evidence validating the causal relationship between LPL subspecies and clinical events. Furthermore, MALDI-MSI has certain limitations in lipid quantification, and processes such as tissue freezing, sectioning, and matrix spraying may affect the *in situ* distribution of lipids; these factors should be considered when interpreting results. Future studies should aim to validate these findings in larger patient cohorts, correlate spatial lipid distribution with clinical outcomes in prospective studies, and develop high-throughput methods to make spatial metabolomics more accessible for clinical diagnostics.

## 6 Breaking the vicious cycle: therapeutic strategies targeting LPLs

The mechanistic studies described above indicate that the lipid-inflammation cycle built by LPLs is a central driver of AS progression. Based on this concept, the key to breaking the cycle is to identify and intervene at its critical nodes. However, translating basic research findings into clinical applications faces substantial hurdles. Single-target blockade frequently fails due to compensatory regulatory mechanisms, indicating that future therapeutic strategies must shift toward multi-node combination interventions.

### 6.1 Upstream node: lowering LDL to reduce the raw material for LPL production

The starting point of the vicious cycle is the excess LDL substrate provided by systemic hyperlipidemia. Therefore, the core goal of upstream intervention is to lower LDL-C levels and reduce ox-LDL generation, thereby cutting LPC and LPA production at the source. Statins, as first-line lipid-lowering therapy, have significantly reduced cardiovascular risk, but the persistence of residual risk indicates that their lipid-lowering effect is still insufficient (4). PCSK9 inhibitors (e.g., evolocumab, alirocumab) enhance LDL receptor recycling and can further lower LDL-C by 50-60%, yet individual benefit varies widely—Kaasenbrood et al. (5) estimated a median lifetime gain of only five months, with 61% of patients deriving less than six months of benefit. This reality suggests that lowering lipids alone cannot fully interrupt the cycle; combination with midstream and downstream interventions is necessary.

### 6.2 Midstream node: blocking LPL signal production and transmission

Midstream interventions target LPL-producing enzymes and their receptors, and are a core part of breaking the pathogenic cycle. However, the successes and failures of clinical trials have revealed just how complex this strategy is.

#### 6.2.1 Lp-PLA₂ inhibitors: divergence between epidemiological signals and clinical trial outcomes

In the circulation, Lp-PLA₂ binds to lipoproteins via ApoB and hydrolyzes oxidized phospholipids in ox-LDL, generating pro-inflammatory lipid species such as lysophospholipids and oxidized free fatty acids (84). Large-scale epidemiological studies consistently showed that circulating Lp-PLA₂ activity is positively associated with cardiovascular event risk, making it a widely investigated therapeutic target (85). On this epidemiological basis, GlaxoSmithKline developed the specific Lp-PLA₂ inhibitor darapladib and launched two large phase III trials (86). Both trials failed. The STABILITY trial (15,828 patients with stable coronary heart disease) and the SOLID-TIMI 52 trial (13,026 patients after acute coronary syndrome) both failed to show significant improvement in the primary composite endpoint (87).

The reasons for darapladib's failure are multiple. First, Mendelian randomization studies suggest that Lp-PLA_2_ itself may be more a marker of inflammation than a causal mediator (88). Second, Lp-PLA_2_ serves a dual role by both generating pro-inflammatory LPC and clearing oxidized phospholipids. For this reason, its inhibition can drive oxidized phospholipid buildup and induce compensatory pathological responses (89). Third, patients enrolled in the trials were already on standard care, had relatively low baseline inflammation, and were not stratified by Lp-PLA_2_ levels, causing “treatment effect dilution”. Fourth, about 20% of patients discontinued due to adverse effects, which weakened statistical power (87, 90). The outcome suggests that the selection of midstream targets must be supported by genetic evidence of causality and take compensatory mechanisms into full account.

#### 6.2.2 ATX inhibitors and LPA receptor antagonists: dual blockade of the signaling axis

Unlike Lp-PLA_2_, the theoretical foundation of the ATX-LPA axis is more solid—ATX is the sole rate-limiting enzyme converting LPC to LPA, and LPA is far more bioactive than LPC. Preclinical studies have shown that ATX inhibitors significantly reduce plaque area and macrophage infiltration in ApoE⁻/⁻ mice (91, 92). However, clinical translation has met setbacks: ziritaxestat (GLPG1690) not only failed to slow the decline in lung function in the phase III ISABELA trials for idiopathic pulmonary fibrosis (IPF), but also showed higher all-cause mortality (93, 94). *post-hoc* analyses revealed that plasma ATX concentrations increased over time, suggesting a compensatory feedback upregulation mechanism (95).

In the AS field, the LPA₁/₃ dual antagonist Ki16425 demonstrated significant efficacy in LDLR⁻/⁻ mice: it reduced lesion area, lowered CCL2 secretion, decreased inflammatory cell infiltration, and increased the proportion of regulatory T cells (96). The LPA₁ antagonist SAR100842 also showed some efficacy in a phase II trial for systemic sclerosis (97). However, it must be emphasized that the clinical evidence for LPA receptor antagonists comes entirely from non-AS diseases (IPF and systemic sclerosis), whose pathophysiological mechanisms differ substantially from atherosclerosis. To date, no LPA receptor antagonist has completed a phase II or III clinical trial specifically for the AS indication. The potential efficacy of Ki16425 and SAR100842 in AS is based solely on preclinical animal model data, and extrapolating from these data to therapeutic benefit in AS patients remains speculative rather than established. This represents a major research gap.

#### 6.2.3 LPA gene-targeted therapy: reducing Lp(a) at the source

Lp(a) is an LDL-like particle composed of apolipoprotein(a) covalently bound to ApoB-100. Lp(a) carries a large amount of oxidized phospholipids and is an important source of LPA and other pro-inflammatory LPLs in the vessel wall. Therefore, targeting the LPA gene to lower Lp(a) levels can be considered a strategy that blocks the LPL signaling axis from an even more upstream position. Globally, more than 20% of the cardiovascular population has elevated Lp(a), yet there are currently no approved pharmacological treatments specifically targeting Lp(a) reduction, and no randomized trial has yet demonstrated that lowering Lp(a) reduces CVD risk (98).

In recent years, antisense oligonucleotides (ASOs) and small interfering RNAs (siRNAs) targeting LPA gene expression have entered clinical development, showing unprecedented reductions in circulating Lp(a) levels. Pelacarsen (ASO) is the most advanced agent, currently being evaluated in the phase III Lp(a)HORIZON trial (8,323 patients) for its effect on cardiovascular outcomes (98). Zerlasiran (siRNA) reduced Lp(a) by 81%–85% in the phase II ALPACAR-360 trial, with median reductions of 94%–96%, was well tolerated, and has advanced to phase III (99). Muvalaplin is the first oral small molecule inhibitor of Lp(a) formation, interfering with Lp(a) assembly. The phase II KRAKEN trial demonstrated that muvalaplin significantly reduced Lp(a) levels by up to 70% (by traditional assay) and up to 85.5% (by a novel isoform-insensitive intact assay), with minimal, dose-independent effects on plasminogen activity (100). Lepodisiran (long-acting siRNA) in the phase II ALPACA trial reduced Lp(a) by 94% after a single injection, with effects lasting over one year (101, 102). A meta-analysis of phase II trials of LPA-targeting siRNAs reported a pooled Lp(a) reduction of 94.5%, accompanied by modest reductions in ApoB and LDL-C, but the incidence of adverse events was higher in the siRNA group and heterogeneity was significant, requiring larger phase III trials to confirm safety (103).

It is important to recognize that the clinical translation of LPA gene-targeted therapies remains in progress. To date, no trial has yet proven that lowering Lp(a) reduces cardiovascular events, and the long-term safety of these agents (particularly their effects on the fibrinolytic system) remains unclear.

### 6.3 Terminal node: inhibiting pyroptosis and inflammatory effects

Even if midstream signals are not fully blocked, terminal interventions may still reduce pathological damage. LPC drives macrophage pyroptosis by activating the NLRP3 inflammasome, a key terminal event in plaque instability (65). NLRP3 inhibitors such as MCC950 have shown anti-inflammatory and anti-AS potential in animal models, but have not yet entered clinical trials for cardiovascular indications (104). Targeting IL-1β represents a highly clinically relevant therapeutic approach. The CANTOS trial demonstrated that canakinumab reduces the risk of cardiovascular events (105). This clinical finding indirectly supports the pathogenic role of the inflammasome–pyroptosis axis in AS, and constitutes the most robust clinical evidence to date for interventions targeting the downstream effectors of LPL signaling.

### 6.4 Future directions: multi-node combination and precise stratification

Looking back at the successes and failures of the clinical trials described above, blocking a single node is easily offset by compensatory mechanisms. The best future strategy is therefore multi-node combination intervention: upstream, reduce the raw material (potent statins or PCSK9 inhibitors to lower LDL-C); midstream, block production (ATX inhibitors to cut LPA generation) and block signaling (LPA_1/3_ receptor antagonists to interrupt pathological transmission); and terminal, block the effect (NLRP3 inhibitors or anti-IL-1β antibodies to suppress pyroptosis and inflammatory amplification). This multi-node combination strategy open aims to break the pathogenic cycle at multiple steps simultaneously.

However, the safety and cost-effectiveness of combination therapy remain major challenges, and responses to treatment may differ significantly between disease stages (early fatty streaks vs. advanced vulnerable plaques) (106). Thus, precise stratification is key to improving the success rate of clinical trials. Spatial metabolomics has shown that LPC (18:0) and LPA (18:1) are highly co-localized in macrophage-rich areas of vulnerable plaques (18), suggesting that LPL candidate biomarkers with specific acyl chains may be more pathogenic. Plasma levels of these specific candidate biomarkers, or their ratios to total LPLs, could become important biomarkers for identifying “LPL-driven” AS patients and enabling precision therapy (18). Future clinical trials should not enroll all AS patients indiscriminately; instead, they should use spatial metabolomics or plasma candidate biomarkers profiles for stratification, targeting interventions only to truly “LPL-driven” patients. This may be the most important lesson learned from the failures of darapladib and ziritaxestat.

Looking back at the successes and failures of the clinical trials described above, blocking a single node is easily offset by compensatory mechanisms. Therefore, a multi-node combination intervention strategy may be optimal: upstream, reduce the raw material (PCSK9 inhibitors to lower LDL-C); midstream, block production (ATX inhibitors to cut LPA generation) and block signaling (LPA₁/₃ receptor antagonists); terminal, block the effect (NLRP3 inhibitors or anti-IL-1β). However, this triple combination strategy faces several practical barriers:

- Additive immunosuppression: anti-IL-1β itself increases the risk of serious infections; combining it with NLRP3 inhibitors or ATX inhibitors may further amplify this risk (105).
- Bleeding and hepatotoxicity: simultaneous inhibition of lipid metabolism and inflammatory pathways may affect platelet function and liver enzymes, but no preclinical data exist to evaluate these risks.
- Cost and adherence: the combination of multiple biologics would significantly increase costs, and frequent injections may severely reduce patient adherence (107).
- Lack of preclinical validation: no AS animal model has yet tested concurrent targeting of upstream, midstream, and terminal nodes; the toxicity or antagonistic effects of such combinations are unknown.

Therefore, clinical translation of multi-node strategies should follow a stepwise validation path: first, test double-node combinations (e.g., PCSK9 inhibitor + ATX inhibitor) in animal models for safety and synergy, then gradually introduce immunomodulatory agents; human trials should adopt adaptive designs and use biomarker-driven patient selection (e.g., plasma LPC/LPA subspecies, hs-CRP) to avoid a “one-size-fits-all” approach. Only after thorough evaluation of safety and efficacy can this theoretically attractive strategy become a viable clinical option.

Furthermore, responses to treatment may differ significantly between disease stages (early fatty streaks vs. advanced vulnerable plaques) (106). Precise stratification is key to improving the success rate of clinical trials. Spatial metabolomics has shown that LPC (18:0) and LPA (18:1) are highly co-localized in macrophage-rich areas of vulnerable plaques (18), suggesting that LPL subspecies with specific acyl chains may be more pathogenic. Plasma levels of these specific subspecies, or their ratios to total LPLs, could become important biomarkers for identifying “LPL-driven” AS patients and enabling precision therapy (18). Future clinical trials should move beyond a “one-size-fits-all” design and instead use spatial metabolomics or plasma subspecies profiles for stratification, targeting interventions only to truly “LPL-driven” patients. This may be the most important lesson learned from the failures of darapladib and ziritaxestat.

 Table 2 summarizes the representative medications, clinical conditions, and major barriers associated with each of the intervention points mentioned above.

**Table 2:** Summary of therapeutic strategies targeting LPLs in atherosclerosis: current status and challenges.

Intervention node	Representative agent/target	Mechanism	Clinical/preclinical status	Major barriers/knowledge gaps
Upstream: reduce substrate	Statins, PCSK9 inhibitors	Lower LDL-C, ox-LDL	Approved, widely used	Residual risk persists
Midstream: block LPL production	Darapladib (Lp-PLA₂ inhibitor)	Inhibits LPC generation	Phase III (failed)	Possibly non-causal target; dual function leads to compensation
	Ziritaxestat (ATX inhibitor)	Blocks LPC→LPA conversion	Phase III (failed in IPF)	Compensatory ATX upregulation; no AS trial
Midstream: block LPL signaling	Ki16425, SAR100842 (LPA receptor antagonists)	Blocks LPA–LPAR binding	Preclinical only (AS); clinical data from non-AS diseases	No AS-specific clinical trial; major gap
Midstream: LPA gene-targeted therapy	Pelacarsen, Zerlasiran, Muvalaplin, Lepodisiran	Reduce Lp(a)	Pelacarsen: phase III ongoing; others: phase II completed	Cardiovascular benefit not yet proven; long-term safety unknown
Terminal: inhibit pyroptosis/inflammation	MCC950 (NLRP3 inhibitor)	Blocks NLRP3 inflammasome	Preclinical	No cardiovascular trial
	Canakinumab (anti-IL-1β)	Neutralizes IL-1β	Phase III positive (CANTOS)	Not LPL-specific; increased infection risk
Multi-node combination	PCSK9 inhibitor + ATX inhibitor + anti-IL-1β	Simultaneously blocks multiple nodes	No preclinical/clinical validation	Immunosuppression, bleeding risk, cost, adherence

AS, atherosclerosis; ATX, autotaxin; CANTOS, Canakinumab Anti-inflammatory Thrombosis Outcomes Study; IL-1β, interleukin-1β; IPF, idiopathic pulmonary fibrosis; LDL-C, low-density lipoprotein cholesterol; Lp(a), lipoprotein(a); Lp-PLA₂, lipoprotein-associated phospholipase A₂; LPA, lysophosphatidic acid; LPAR, lysophosphatidic acid receptor; LPC, lysophosphatidylcholine; LPLs, lysophospholipids; NLRP3, NLR family pyrin domain containing 3; ox-LDL, oxidized low-density lipoprotein; PCSK9, proprotein convertase subtilisin/kexin type 9. For LPA gene-targeted therapies, no trial has yet demonstrated cardiovascular event reduction, and long-term safety (e.g., effects on the fibrinolytic system) remains under evaluation. Pelacarsen is currently being investigated in the phase III Lp(a)HORIZON trial (NCT04023552); zerlasiran (ALPACAR-360), muvalaplin (KRAKEN), and lepodisiran (ALPACA) have completed phase II and are advancing toward phase III. The multi-node combination strategy lacks preclinical and clinical validation, and the listed barriers are based on theoretical considerations rather than empirical evidence.

## 7 Discussion and conclusion

### 7.1 Integration of key findings and theoretical implications

This review takes the “lipid-inflammation cycle” as its central logical thread and systematically elaborates the hub role of lysophospholipids (LPLs) in atherosclerosis (AS). This framework does not reject the earlier “lipid infiltration” or “inflammation” hypotheses; rather, it attempts to integrate them—LPLs possess both lipid-signal and inflammatory-mediator properties, placing them exactly at the intersection of the two theories. Studies by Li et al. (41) and Schober et al. (61) laid the foundation for the LPL signaling network in AS, while the discovery by Corrêa et al. (65) that LPC triggers NLRP3 pyroptosis provided a concrete molecular example of how lipids translate into inflammatory injury. Spatial metabolomics evidence (18) has further advanced this mechanism from cell culture to tissue *in situ*, providing multi-level evidence from molecular to tissue scales from the molecular to the tissue level.

However, this “pathogenic cycle” framework still has theoretical boundaries. First, multiple endogenous pathways act to counteract LPL-mediated injury: high-density lipoprotein (HDL) sequesters and clears LPC, and liver X receptor (LXR) signaling induces ABCA1 to drive cholesterol efflux (108, 109). How these negative feedback regulations interact with the self-reinforcing pathogenic center remains unclear. Second, not all hyperlipidemic patients develop AS; factors such as genetic background, baseline inflammation levels, and gut microbiota composition may determine the threshold for triggering the cycle, but research in this area is still at an early stage.

### 7.2 Challenges and reflections on clinical translation

The path from basic research to clinical application has not been smooth. The failure of the Lp-PLA₂ inhibitor darapladib (87, 88, 90) and the setback of the ATX inhibitor ziritaxestat in IPF (94, 95) together reveal deep-seated difficulties in cardiovascular drug development.

First, insufficient validation of target causality. Mendelian randomization studies can partially address confounding in observational studies, but their predictive value for complex diseases such as AS is limited (88). AS results from the interplay of multiple genes and environmental factors; genetic validation of a single target in such a complex setting cannot fully predict its clinical efficacy (88).

Second, the complexity of compensatory mechanisms has been underestimated. The dual function of Lp-PLA_2_ (generating pro-inflammatory LPC vs. clearing oxidized phospholipids) and the feedback upregulation of the ATX-LPA axis (95) indicate that signaling networks under chronic pathological conditions possess substantial adaptive redundancy. Future target assessment should incorporate systems biology approaches to model network reconfiguration after long-term inhibition (110, 111).

Third, the lack of patient stratification strategies. The STABILITY and SOLID-TIMI 52 trials did not stratify patients by baseline Lp-PLA₂ levels or inflammatory phenotypes, leading to “treatment effect dilution”. The success of the CANTOS trial can be partly attributed to its hs-CRP-based patient selection, which offers an important lesson for LPL-directed therapy: future trials should stratify patients according to plasma LPL subspecies profiles or spatial metabolomic features, and enrol only those who are truly “LPL-driven”.

Pre-specified subgroup analyses of the STABILITY and SOLID-TIMI 52 trials failed to identify any patient population that benefited from darapladib; whether by baseline Lp-PLA₂ activity level or by the degree of on-treatment Lp-PLA₂ inhibition, darapladib did not significantly improve the primary endpoint (90, 112, 113). This suggests that both trials may have suffered from “treatment effect dilution” because patients were not stratified by inflammatory phenotype. In stark contrast, the CANTOS trial enrolled patients with residual inflammatory risk (hs-CRP ≥2 mg/L) and successfully demonstrated clinical benefit from targeting the inflammatory pathway; subsequent analysis showed a clear dose-dependent relationship between the extent of hs-CRP reduction and the reduction in cardiovascular events (114). The success of CANTOS offers a crucial lesson for LPL-directed therapy: future clinical trials should abandon a “one-size-fits-all” design and instead use plasma LPL subspecies profiles or spatial metabolomic features to enroll only truly “LPL-driven” patients. Only then can we hope to overcome the translational hurdles that have thwarted Lp-PLA_2_ and ATX targeting so far.

### 7.3 Future research directions

Based on the above analysis, future research should focus on the following three areas:

First, clarifying the dual pro-inflammatory and anti-inflammatory nature of LPC. Current studies have focused mainly on the pro-inflammatory effects of LPC, but could LPC also have pro-resolving functions at certain concentrations or in specific microenvironments? Work by Wang et al. (46) and Tontonoz et al. (47) on LPC-PPAR*γ* interactions suggests that PPAR*γ* activation can both promote inflammation (M1 polarization) and induce lipid metabolic reprogramming. This duality is critically important for the safety assessment of any targeting strategy.

Second, developing selective targeting technologies for specific LPL subspecies. Spatial metabolomics has identified LPC (18:0) and LPA (18:1) as key pathogenic subspecies (18), but existing drugs (e.g., darapladib, ziritaxestat) are broad-spectrum inhibitors that cannot distinguish subspecies with different acyl chain compositions. Developing antibodies or small molecules that selectively target based on fatty acid chain length or saturation is a technical prerequisite for precision intervention.

Third, the interaction between LPLs and the gut microbiota. The gut microbiota can produce various phospholipid metabolites. Do these metabolites enter the circulation and affect the LPL pool in the vessel wall? Current evidence indicates that gut dysbiosis plays an important role in the development of atherosclerosis (115), and the “gut-heart axis” hypothesis has been widely validated with microbial metabolites such as TMAO, LPS, and SCFAs in regulating AS inflammation (115). However, the causal chain from microbial phospholipid metabolism directly to the vascular wall LPL pool, i.e., whether dietary phosphatidylcholine is converted by the gut microbiota into LPC/LPA, enters the circulation, and subsequently modulates the vascular LPC/LPA pool, has not yet been directly tested in the AS field. This hypothesis may open an entirely new mechanistic perspective on the link between diet, microbiota, and cardiovascular risk.

## 8 Conclusions

In summary, LPLs act as key hub messengers linking lipid metabolism disorders and chronic inflammation in AS. Through a self-reinforcing vicious cycle of “production-amplification-feedback”, they drive plaque progression and destabilization. Moving from basic mechanisms to clinical translation requires moving beyond linear thinking focused on single targets and shifting toward multi-node combination interventions and precision therapy based on molecular subtyping. Targeting LPLs may offer a new way to overcome the “residual risk” of AS, a major public health challenge. But achieve this goal will need not only deeper mechanistic understanding, but also innovative translational thinking that turns our knowledge of molecular pathology into effective therapies that truly improve patient outcomes.
